# Supplementary material for: El Niño-like teleconnection increases California precipitation in response to warming
Source: Nat Commun. 2017 Jul 6;8:16055. doi: 10.1038/ncomms16055 (PMC5504297; doi:10.1038/ncomms16055)
Supplement: Supplementary Information [file ncomms16055-s1.pdf]

Title of file for HTML: Supplementary Information
Description: Supplementary Figures, Supplementary Tables, Supplementary Discussio
n and Supplementary Reference

Title of file for HTML: Peer Review File
Description:

Supplementary Discussion

Alternative Correlation Thresholds for CMIP5 HIGH−r and LOW−r Modifying the Niño 3.4 SST versus CA precipitation correlation thresholds used to define CMIP5 HIGH−r and LOW−r models yields similar conclusions. For example, a threshold of 0.40 for CMIP5 HIGH−r yields 7 models (17 realizations in total) and an ensemble mean increase in DJF (ANN) CA precipitation of 0.76 (0.15) mm day $^{-1}$ century^{-1}, both significant at the 99% confidence level. For both DJF and ANN, 71% of the realizations yield an increase in CA precipitation. Using a threshold of 0.10 for CMIP5 LOW−r yields 8 models (11 realizations in total) and an ensemble mean decrease in DJF (ANN) CA precipitation of−0.08 (−0.22) mm day $^{-1}$ century^{-1}, with the ANN trend significant at the 99% confidence level. 45% (18%) of the realizations yield an increase in DJF (ANN) CA precipitation.

Similar conclusions are also obtained if we choose thresholds at which the Niño 3.4 SST and CA precipitation correlation first becomes significant at the 99% (not significant at the 90%) confidence level for CMIP5 HIGH−r (CMIP5 LOW−r). We note that these thresholds, at 0.27 and 0.16 respectively, are nearly identical to the default thresholds. The corresponding CMIP5 LOW−r results are nearly identical to the original results, since only one model-realization (GFDL-ESM2M) is removed from the original CMIP5 LOW−r subset. The corresponding CMIP5 HIGH−r subset yields 16 models and 33 realizations (an addi-tional 2 models and 4 realizations relative to the original CMIP5 HIGH−r subset) and an ensemble mean increase in DJF (ANN) CA precipitation of 0.72 (0.12) mm day $^{-1}$ century^{-1}, both significant at the 99% confidence level. 79% (67%) of the realizations yield an increase

in DJF (ANN) CA precipitation. Thus, these alternate thresholds also suggest that models that better reproduce the observed Niño 3.4 SST and CA precipitation teleconnection tend to yield larger, and more consistent, increases in CA precipitation through the 21st century.

Alternative Methodologies for CMIP5 HIGH−r and LOW−r

High correlation between CA precipitation and Niño 3.4 SSTs alone does not guarantee a more realistic response. Supplementary Table 2 shows late-20th century (1948/49-2004/05) DJF correlations, regression coefficients, and climatological CA precipitation by region for CMIP5 models, along with the observed values and their 1-sigma uncertainty. Similar to Langenbrunner and Neelin (2013), the CA precipitation sensitivity to Niño 3.4 SSTs, and regional climatologies, for many models falls outside the observed range. This includes CESM1-CAM5 and GFDL-CM3, with precipitation sensitivities of 0.71 and 0.91 mm day^{-1} per °C, respectively. CESM1-CAM5 also exceeds the upper bound of the observed range of CA DJF precipitation for the entire state, as well as central and northern CA, by 0.4, 0.5 and 0.7 mm day^{-1}, respectively. GFDL-CM3 also exceeds the upper bound of the observed range of CA precipitation for all regions, with a 1.4 mm day^{-1} overestimate for the entire state.

If we use the observed CA precipitation versus Niño 3.4 SST regression coefficient (±1-sigma), "HIGH−r" ("LOW−r") models are defined as those with a regression coefficient between 0.23 and 0.61 (less than 0.23) mm day^{-1} per °C. This results in 12 HIGH−r models with 34 realizations, and 18 LOW−r models with 32 realizations. The ensemble mean 21st century DJF (ANN) CA precipitation trend in these HIGH−r models is 0.64 (0.11) mm day^{-1} century^{-1}, both significant at the 99% confidence level. 80% (71%) of the realizations yield an

increase in DJF (ANN) CA precipitation. The corresponding DJF (ANN) CA precipitation trend for LOW−r models is 0.37 (−0.01), the former significant at the 99% confidence level. 66% (41%) of the realizations yield an increase in DJF (ANN) CA precipitation. Thus, using this alternate methodology, we obtain similar conclusions: HIGH−r models simulate a larger, and more consistent increase in 21st century CA precipitation.

Finally, we define a "HIGH−r" model subset the uses all of the above criteria. This includes 1. late-20th and 21st century correlations between DJF CA precipitation and Niño 3.4 SSTs that are significant at the 90% confidence level; 2. late-20th century DJF CA precipitation versus Niño 3.4 SST regression coefficient that falls within 1-sigma of the observed range (0.23 to 0.61 mm day^{-1} per °C); 3. late-20th century DJF CA precipitation climatologies that fall within 1-sigma of the observed range. This latter criterion equates to 1.6 to 3.6 mm day^{-1} for CA; 0.5 to 2.3 mm day^{-1} for southern CA; 1.2 to 3.3 mm day^{-1} for central CA; and 2.5 to 5.9 mm day^{-1} for northern CA.

Using these three criteria, we obtain three models with 12 realizations−IPSL-CM5A-LR, CanESM2 and FIO-ESM. The ensemble mean 21st century DJF (ANN) CA precipitation trend in these HIGH−r models is 0.91 (0.24) mm day^{-1} $century^{-1}$, both significant at the 99% confidence level. 83% (83%) of the realizations yield an increase in DJF (ANN) CA precipitation. These values are very similar to those based on our original correlation-based threshold. There, the ensemble mean 21st century DJF (ANN) CA precipitation trend in HIGH−r models is 0.84 (0.16) mm day^{-1} $century^{-1}$, both significant at the 99% confidence level. 79% (72%) of the realizations yield an increase in DJF (ANN) CA precipitation. Moreover, the tropical and extratropical dynamical responses in these three models is similar to that discussed in the manuscript. Thus, we continue to find significant and robust increases

in 21st century CA precipitation.

CMIP5 California Precipitation Trends Using Alternative RCPs

We obtain similar conclusions based on the CMIP5 models, but using other RCPs. Using our original correlation thresholds and CMIP5 RCP 6.0 data, CMIP5 HIGH−r yields 6 models (15 realizations in total) and an ensemble mean increase in DJF (ANN) CA precipitation of 0.68 (0.18) mm day $^{-1}$ century^{-1}, both significant at the 99% confidence level (Supplementary Figure 4). 87% (73%) of the realizations yield an increase in DJF (ANN) CA precipitation. CMIP5 LOW−r yields 9 models (20 realizations in total) and an ensemble mean increase in DJF (ANN) CA precipitation of 0.09 (0.02) mm day $^{-1}$ century^{-1}, neither significant at the 90% confidence level. 50% (60%) of the realizations yield an increase in DJF (ANN) CA precipitation.

Based on RCP 4.5, CMIP5 HIGH−r yields 11 models (26 realizations in total) and an ensemble mean increase in DJF (ANN) CA precipitation of 0.41 (0.10) mm day $^{-1}$ century^{-1}, significant at the 99% and 95% confidence level, respectively. For both DJF and ANN, 77% of the realizations yield an increase in CA precipitation. CMIP5 LOW−r yields 15 models (34 realizations in total) and an ensemble mean increase in DJF (ANN) CA precipitation of 0.21 (0.04) mm day $^{-1}$ century^{-1}, with the DJF trend significant at the 90% confidence level. 59% (53%) of the realizations yield an increase in DJF (ANN) CA precipitation.

Supplementary Table 1 **Coupled Model Intercomparison Project version 5 models and number of simulations used for each Representative Concentration Pathway 8.5, 1% CO_2, and atmosphere-only experiment.** Also included is the 2006-2100 RCP8.5 detrended DJF correlation (r) between Niño 3.4 sea surface temperature and California precipitation. The corresponding correlation based on NOAA's Precipitation Reconstruction from 1948/49 to 2014/15 is 0.36, significant at the 99% confidence level. Based on GPCP data over 1979/80 to 2014/15, the correlation is 0.40, significant at the 95% confidence level. This information was used to determine CMIP5 HIGH−r and LOW−r models. Atmosphere-only experiments include AMIP, AMIP4K and AMIP Future. For the 1% CO_2 experiments, years 1-140 are analyzed.

Institution	Model	AMIP	1% CO_2	RCP8.5	r
CSIRO and Bureau of Meteorology	ACCESS1.0	0	1	1	0.22
	ACCESS1.3	0	1	1	0.22
Beijing Climate Center	BCC-CSM1.1	1	1	1	0.06
	BCC-CSM1.1(m)	0	1	1	0.31
GCESS, Beijing Normal University	BNU-ESM	0	1	1	0.43
Canadian Centre for Climate Modelling and Analysis	CanESM2	1	1	5	0.44
National Center for Atmospheric Research	CCSM4	1	1	5	0.42
Community Earth System Model Contributors	CESM1(BGC)	0	1	1	0.50
	CESM1(CAM5)	0	1	3	0.32
	CESM1-CAM5-1-FV2	0	1	0	n/a
Centro Euro-Mediterraneo per I Cambiamenti Climatici	CMCC-CM	0	1	1	0.30
	CMCC-CMS	0	0	1	0.33
CNRM/CERFACS	CNRM-CM5	1	1	5	0.20
CSIRO, Industrial Research Organization & QCCCE	CSIRO-Mk3.6.0	0	1	10	0.20
EC-EARTH consortium	EC-EARTH	0	0	4	0.08
LASG, IAP, Chinese Academy of Sciences and CESS	FGOALS-g2	0	1	1	0.27
	FGOALS-s2	0	1	0	n/a
The First Institute of Oceanography, SOA, China	FIO-ESM	0	0	3	0.23
NOAA Geophysical Fluid Dynamics Laboratory	GFDL-CM3	0	1	1	0.58
	GFDL-ESM2G	0	1	1	0.12
	GFDL-ESM2M	0	1	1	0.17
NASA Goddard Institute for Space Studies	GISS-E2-R	0	1	1	0.01
	GISS-E2-R_p2	0	1	0	n/a
	GISS-E2-R_p3	0	1	1	0.10
	GISS-E2-H	0	1	1	0.06
	GISS-E2-H_p2	0	1	0	n/a
	GISS-E2-H_p3	0	1	1	−0.08
Met Office Hadley Centre	HadGEM2-ES	0	1	4	0.20
	HadGEM2-AO	0	0	1	0.14
	HadGEM2-CC	0	0	3	0.16
	HadGEM2-A	1	0	0	n/a
Institute for Numerical Mathematics	INM-CM4	0	1	1	0.26
Institut Pierre-Simon Laplace	IPSL-CM5A-LR	1	1	4	0.31
	IPSL-CM5A-MR	0	1	1	0.30
	IPSL-CM5B-LR	1	1	1	0.37
JAMEST, AORI, and NIES	MIROC-ESM	0	1	1	−0.12
	MIROC-ESM-CHEM	0	0	1	−0.03
AORI, NIES, and JAMEST	MIROC5	1	1	3	0.51
Max Planck Institute for Meteorology	MPI-ESM-LR	1	1	3	0.28
	MPI-ESM-MR	1	1	1	0.23
	MPI-ESM-P	0	1	0	n/a
Meteorological Research Institute	MRI-CGCM3	1	1	1	0.40
Norwegian Climate Centre	NorESM1-M	0	1	1	0.22
	NorESM1-ME	0	1	0	n/a

Supplementary Table 2 **Coupled Model Intercomparison Project version 5 late 20th century (1948/49-2004/05) California precipitation statistics.** Included is the detrended December-January-February (DJF) correlation (r) and regression slope (m; mm day^{-1} per °C) between Niño 3.4 sea surface temperature and California precipitation, as well as the climatological CA DJF precipitation (mm day^{-1}) for the entire state, as well as southern (32.0-34.9°N; 239.4-245.6°E), central (34.9-38.6°N; 236.9-243.1°E) and northern (38.8-42.4°N; 235.6-240.6°E) CA regions only. The corresponding statistics based on NOAA's Precipitation Reconstruction (PREC) from 1948/49 to 2014/15, and Global Precipitation Climatology Project (GPCP) over 1979/80 to 2014/15, including the 1-sigma uncertainty, are also listed.

Data/Model	m	r	CA	Southern CA	Central CA	Northern CA
PREC	0.34±0.11	0.36	2.5±0.9	1.2±0.7	2.3±1.0	3.9±1.4
GPCP	0.42±0.19	0.40	2.6±1.0	1.4±0.9	2.2±1.0	4.3±1.6
ACCESS1.0	0.26	0.15	3.6	1.8	3.3	5.8
ACCESS1.3	0.11	0.06	3.3	1.4	3.1	5.5
BCC-CSM1.1	−0.08	−0.05	3.5	2.4	3.4	4.6
BCC-CSM1.1(m)	0.15	0.11	4.9	2.6	5.0	6.9
BNU-ESM	0.21	0.27	3.2	1.5	3.1	5.3
CanESM2	0.23	0.29	2.3	0.7	2.0	4.4
CCSM4	0.55	0.43	3.7	1.5	3.4	6.3
CESM1(BGC)	0.61	0.45	3.6	1.5	3.3	6.2
CESM1(CAM5)	0.71	0.48	4.0	1.7	3.8	6.6
CMCC-CM	0.02	0.01	4.8	2.9	5.1	6.5
CMCC-CMS	0.80	0.49	5.0	2.9	5.2	6.9
CNRM-CM5	0.19	0.15	3.4	1.3	3.0	6.2
CSIRO-Mk3.6.0	0.33	0.16	3.3	1.5	3.4	5.0
EC-EARTH	−0.22	−0.11	3.7	1.8	3.4	5.9
FGOALS-g2	0.27	0.18	4.0	2.1	3.9	6.0
FIO-ESM	0.28	0.34	3.0	1.5	2.8	4.8
GFDL-CM3	0.91	0.55	5.0	2.9	5.1	6.9
GFDL-ESM2G	0.18	0.14	3.9	1.6	3.9	6.1
GFDL-ESM2M	0.22	0.27	4.3	2.0	4.3	6.6
GISS-E2-R	0.24	0.13	5.0	2.5	5.0	7.6
GISS-E2-R_p3	0.07	0.03	4.7	2.5	4.7	7.1
GISS-E2-H	0.28	0.11	5.3	3.1	4.9	7.8
GISS-E2-H_p3	0.32	0.15	4.7	2.5	4.7	7.1
HadGEM2-ES	0.19	0.15	2.4	1.2	2.1	4.2
HadGEM2-AO	0.30	0.24	2.5	1.3	2.2	4.2
HadGEM2-CC	−0.07	−0.05	2.9	1.7	2.6	4.4
INM-CM4	0.12	0.06	3.1	1.2	2.7	5.5
IPSL-CM5A-LR	0.51	0.27	3.4	1.7	3.2	5.4
IPSL-CM5A-MR	1.12	0.56	3.8	1.5	3.7	6.2
IPSL-CM5B-LR	1.31	0.47	6.5	3.9	6.9	8.8
MIROC-ESM	−0.64	−0.34	3.2	1.3	2.7	5.7
MIROC-ESM-CHEM	−0.01	−0.01	3.1	1.2	2.5	5.6
MIROC5	0.67	0.55	3.9	2.0	3.8	6.0
MPI-ESM-LR	0.16	0.11	4.2	1.7	4.1	6.9
MPI-ESM-MR	−0.08	−0.04	4.4	1.7	4.3	7.2
MRI-CGCM3	1.39	0.42	6.2	3.2	6.3	9.3
NorESM1-M	0.21	0.19	2.6	1.3	2.4	4.3

Supplementary Table 3 **21st century ensemble mean December-January-February Community Earth System Model Large Ensemble moisture/dynamical statistics**. All values are significant at the 99% confidence level unless denoted with bold. Interannual correlations are based on the detrended ensemble mean time series (similar results are generally obtained based on the ensemble mean correlation).

Variable	Trend	Trend Correlation w/ CA Precip	Interannual Correlation w/ CA Precip
CA Precip	0.94	X	X
CA MFC	0.69	0.98	0.99
CA MFC Mean	0.72	0.79	0.95
CA MFC Transient	**−0.04**	**−0.07**	**0.30**
CA MFC Mean Dynamic	0.80	0.87	0.97
CA MFC Mean Thermodynamic	**−0.10**	**0.02**	**−0.05**
East Pacific U300	3.65	0.61	0.89
East Pacific *pp*	0.70	0.49	0.85
Niño 3.4 SSTs	4.26	**0.21**[a]	0.62

Trend units are mm day^{-1} century^{-1} for Precip and MFC; m s^{-1} century^{-1} for U300; hPa^2 century^{-1} for *pp*; and K century^{-1} for Niño 3.4.

[a] Based on Figure 3 from the manuscript, a significant trend correlation between CESM LENS tropical central/eastern Pacific SSTs and CA precipitation exists. However, it is shifted slightly westward of the Niño3.4 SST region. Shifting the Niño 3.4 SST region westward 15° (175-225E) yields a trend correlation of 0.33, significant at the 95% confidence level. Note that recalculating all of our correlations that involve Niño 3.4 SSTs, but using this westward shifted SST region, yields nearly identical results (i.e., the ENSO teleconnection is unchanged using 190-240E or 175-225E). Based on CMIP5 HIGH−r models, the trend correlation between Niño 3.4 SSTs and CA precipitation is 0.35, significant at the 95% confidence level. A similar correlation is obtained using the westward shifted Niño 3.4 SST region.

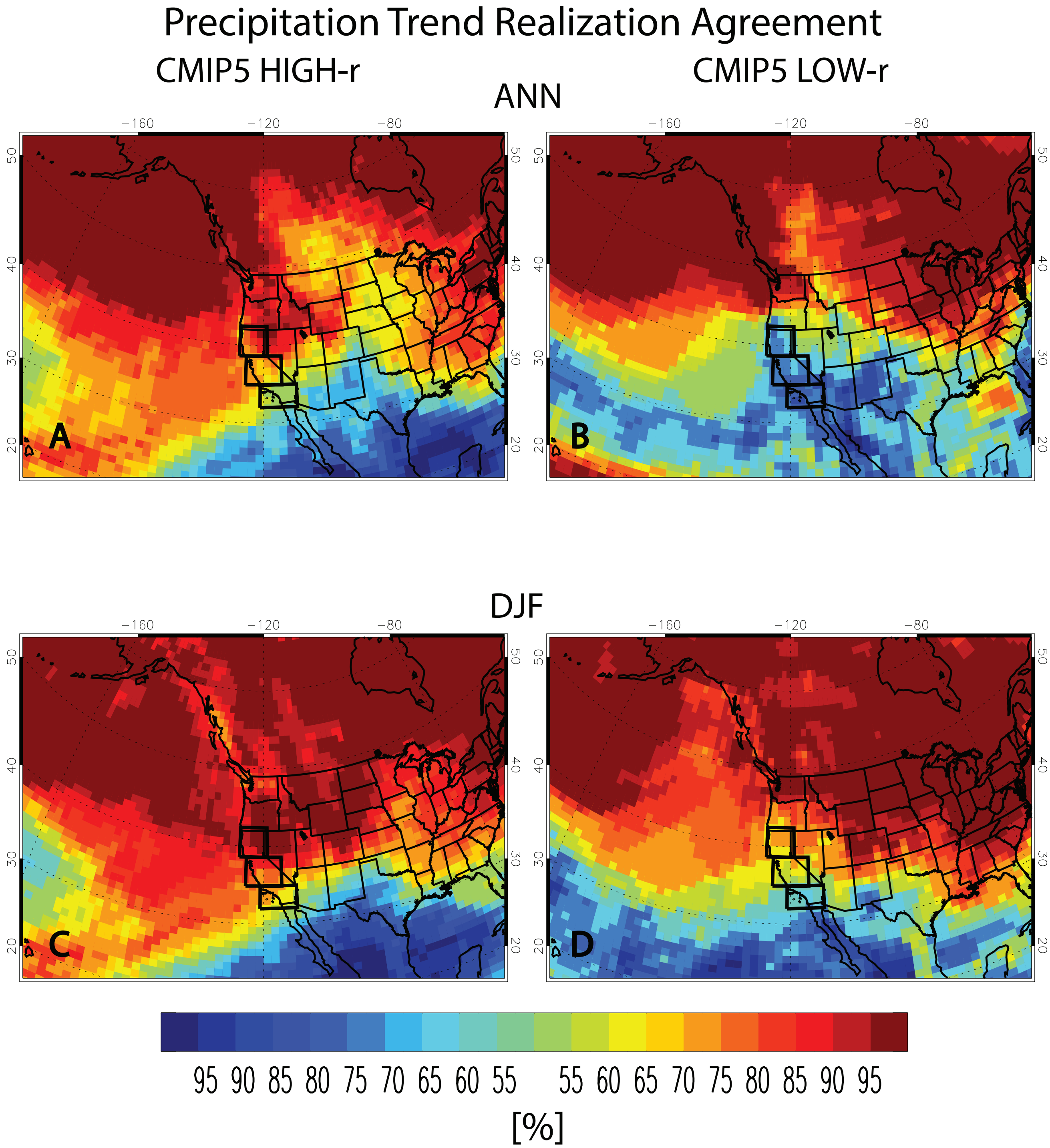

Supplementary Figure 1 **Coupled Model Intercomparison Project version 5 2006-2100 precipitation trend realization agreement.** (A, B) Annual (ANN) and (C,D) December-January-February (DJF) trend realization agreement for two CMIP5 model subsets. Left panels show the model subset that yield a detrended DJF Niño 3.4 SST versus CA precipitation correlation of at least 0.30 (CMIP5 HIGH−r); Right panels show the model subset that yield a corresponding correlation less than 0.20 (CMIP5 LOW−r). Warm (cold) colors show the percent of realizations that yield an increase (decrease) in precipitation.

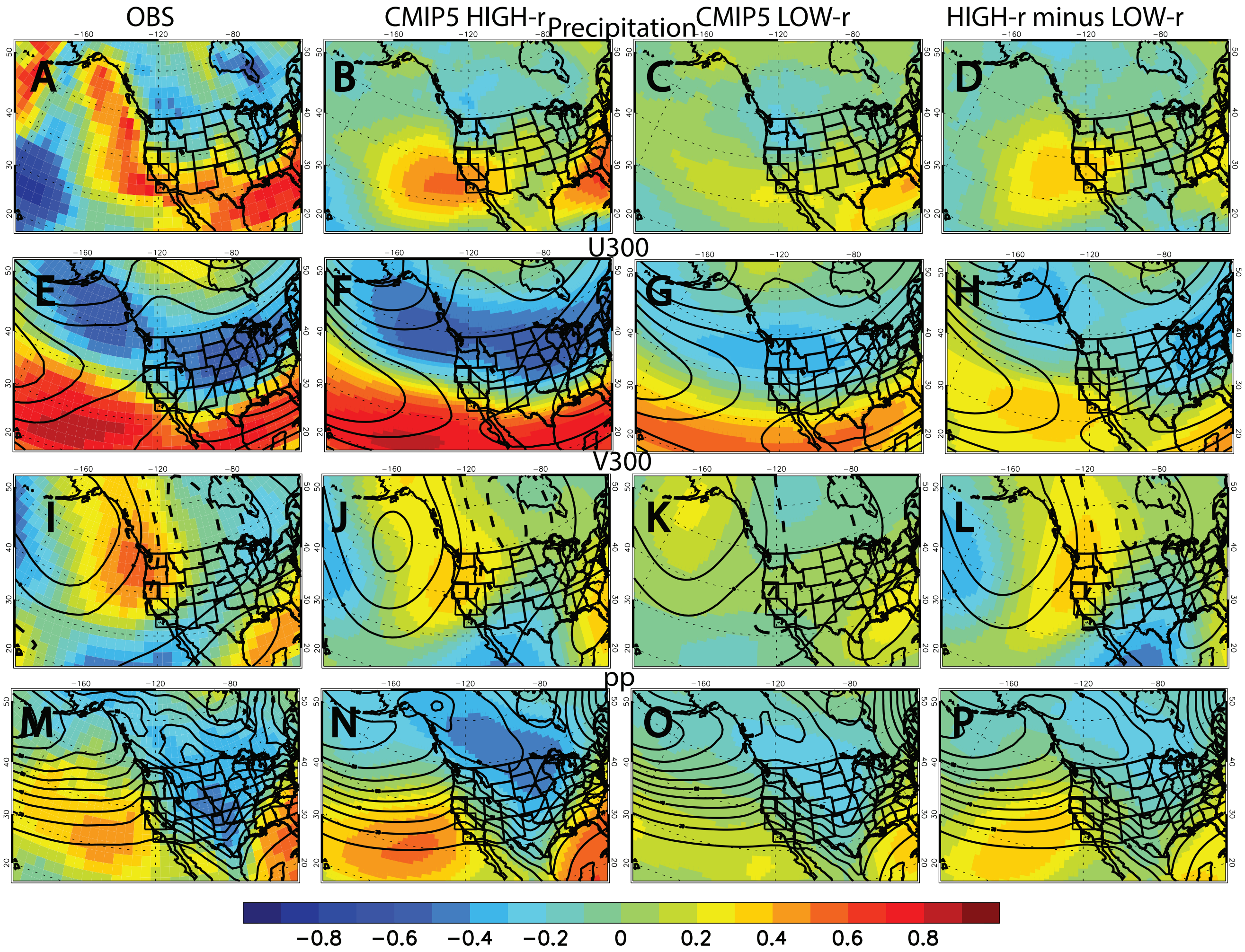

SUPPLEMENTARY FIGURE 2 **Coupled Model Intercomparison Project version 5 detrended December-January-February extratropical correlations with Niño 3.4 SSTs.** (A-D) Precipitation; (E-H) U300; (I-L) V300; and (M-P) *pp* for (left panels) observations and CMIP5 (center left panels) HIGH−r models; (center right panels) LOW−r models; and (right panels) HIGH−r minus LOW−r models. Observations include NCEP/NCAR reanalysis data and NOAA's Precipitation Reconstruction Dataset from 1948 to 2015.

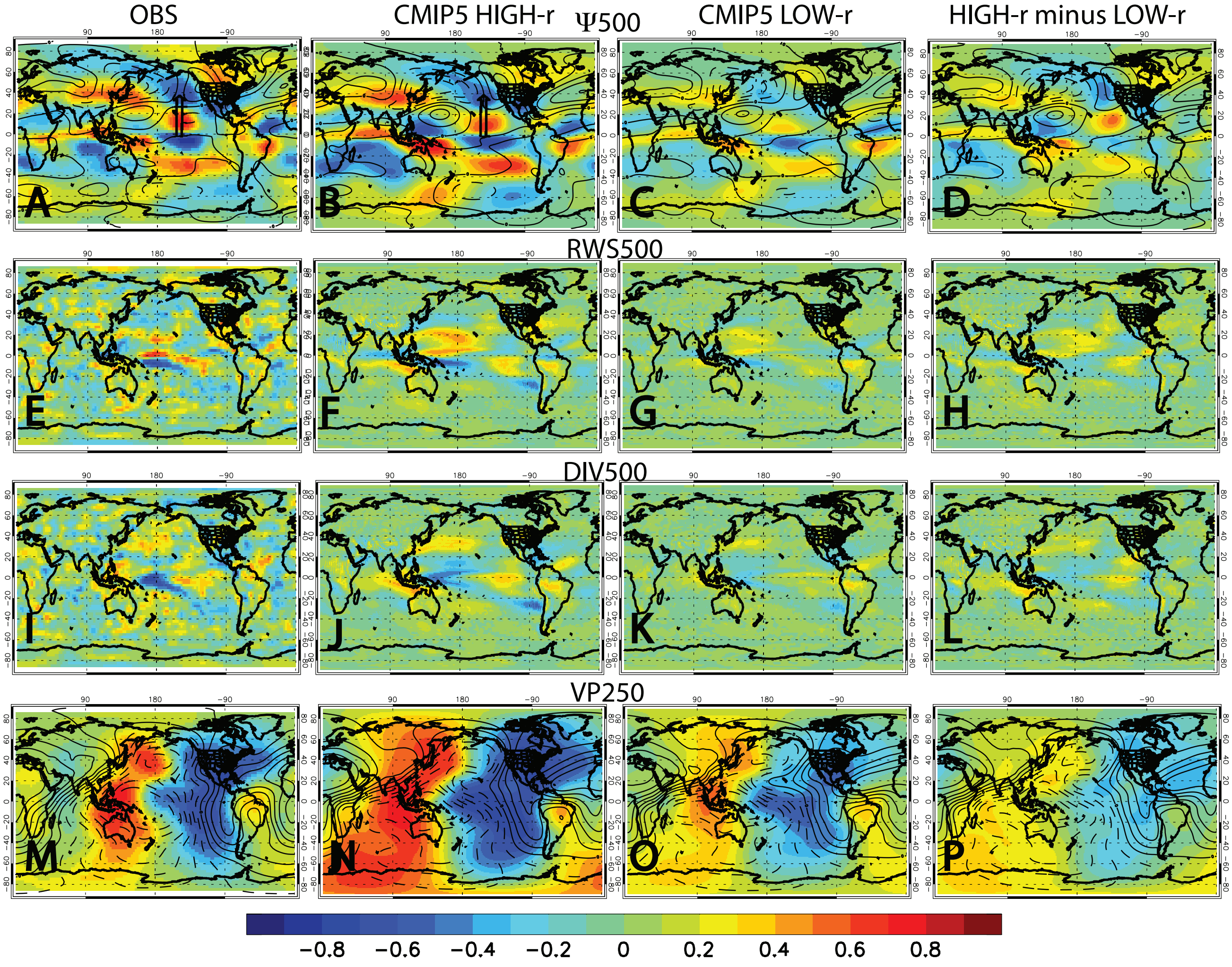

Supplementary Figure 3 **Coupled Model Intercomparison Project version 5 detrended DJF tropical correlations with Niño 3.4 sea surface temperatures.** (A-D) stationary eddy stream function (Ψ500 ; calculated as the deviation from the zonal time mean); (E-H) Rossby wave source at 500 hPa (RWS500); (I-L) divergence at 500 hPa (DIV500); and (M-P) velocity potential at 250 hPa (VP250) for (left panels) observations and CMIP5 (center left panels) HIGH−r models; (center right panels) LOW−r models; and (right panels) HIGH−r minus LOW−r models. Observations include NCEP/NCAR reanalysis data and NOAA's Precipitation Reconstruction Dataset from 1948 to 2015. Black arrows sketch the direction in which the Rossby wave propagates in the Northern Hemisphere.

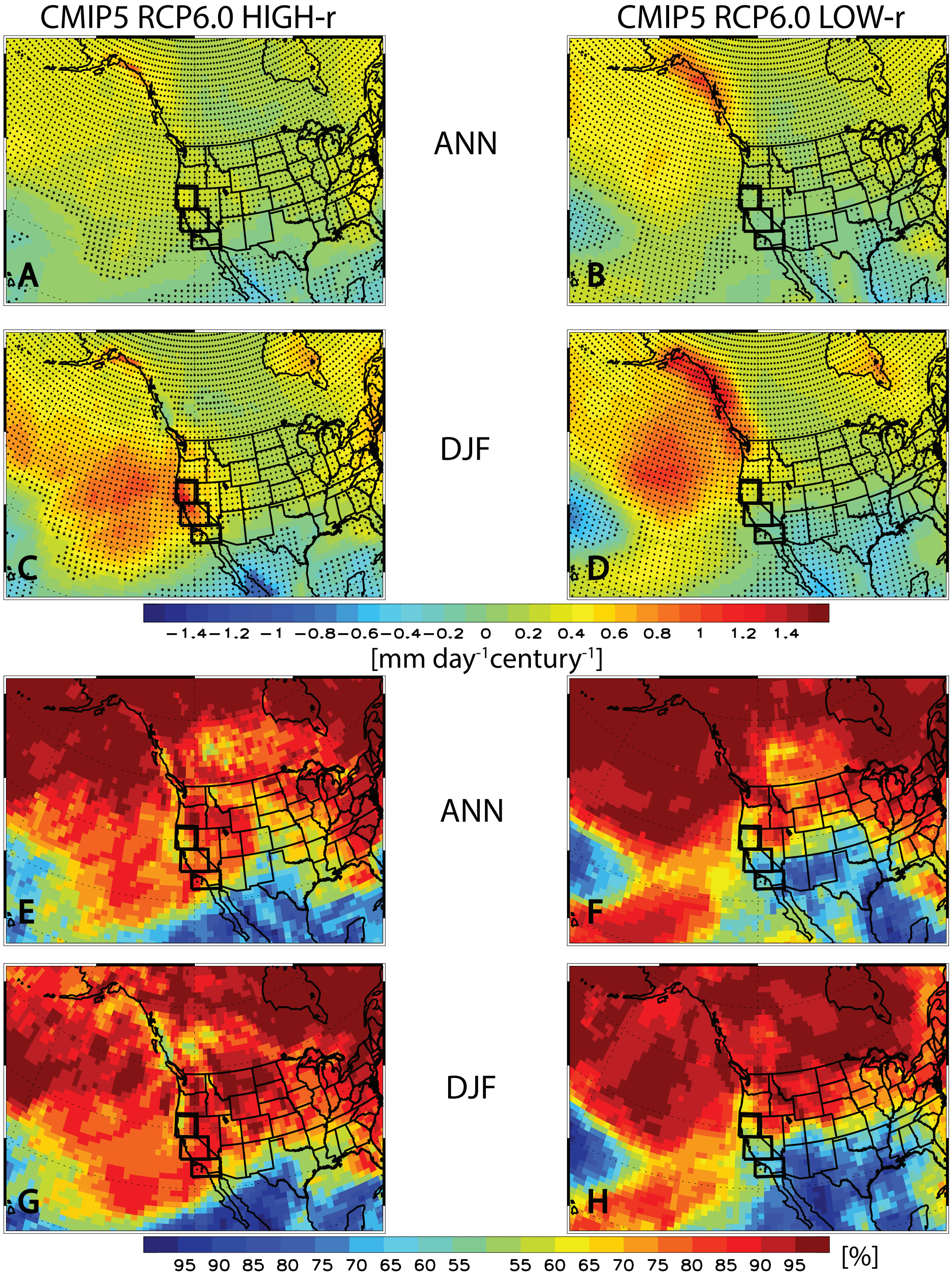

Supplementary Figure 4 **Coupled Model Intercomparison Project version 5 2006-2100 precipitation trend and realization agreement for Representative Concentration Pathway 6.0.** (A, B) Annual (ANN) and (C, D) December-January-February (DJF) ensemble mean precipitation trend [mm day^{-1} century^{-1}]; (E, F) ANN and (G, H) DJF precipitation trend realization agreement [%]. Left panels show the CMIP5 RCP6.0 model subset that yield a detrended DJF Niño 3.4 SST versus CA precipitation correlation of at least 0.30 (CMIP5 HIGH−r); Right panels show the CMIP5 RCP6.0 model subset that yield a corresponding correlation less than 0.20 (CMIP5 LOW−r). Symbols in A-D represent trend significance at the 90% (diamond), 95% (X) or 99% (+) confidence level, accounting for autocorrelation.

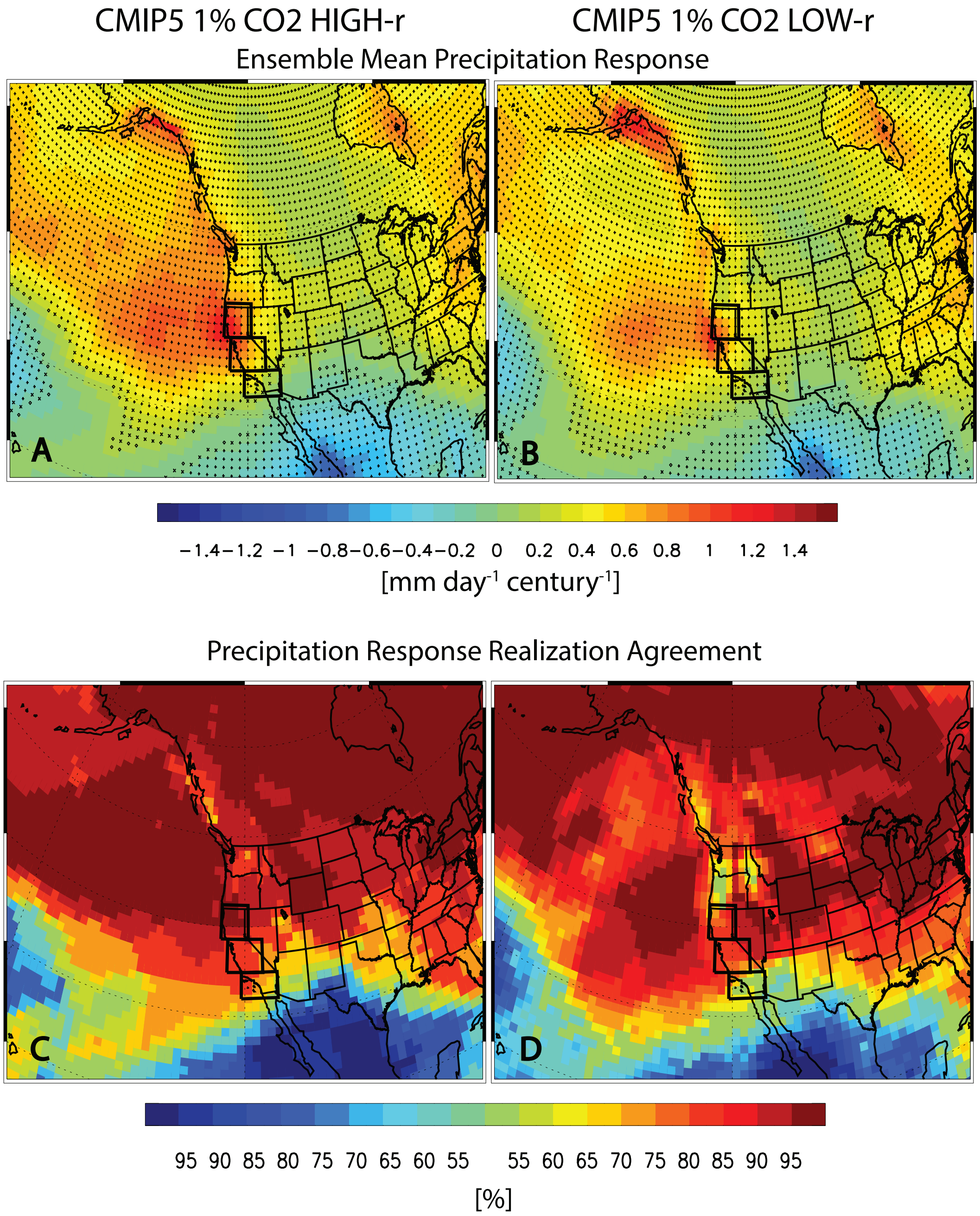

Supplementary Figure 5 **California precipitation response in 1% CO_2 simulations.** (A, B) Ensemble mean (left panels) CMIP5 HIGH−r and (right panels) CMIP5 LOW−r precipitation response [mm day^{-1}] in 1% CO_2 experiments. (C, D) shows the precipitation response realization agreement [%]. Symbols in (A, B) represent trend significance at the 90% (diamond), 95% (X) or 99% (+) confidence level, accounting for autocorrelation. Trends are based on years 1-140, where the last year corresponds to the time of CO_2 quadrupling. CMIP5 1% CO_2 HIGH−r (LOW−*r*) models yield an ensemble mean DJF CA precipitation increase of 0.63 (0.47) mm day^{-1}, both significant at the 99% confidence level, with 83% of the HIGH−r models yielding an increase. Corresponding ANN CA precipitation trends are 0.12 (0.05) mm day^{-1}, both significant at the 99% confidence level, with 75% of the HIGH−r models yielding an increase.

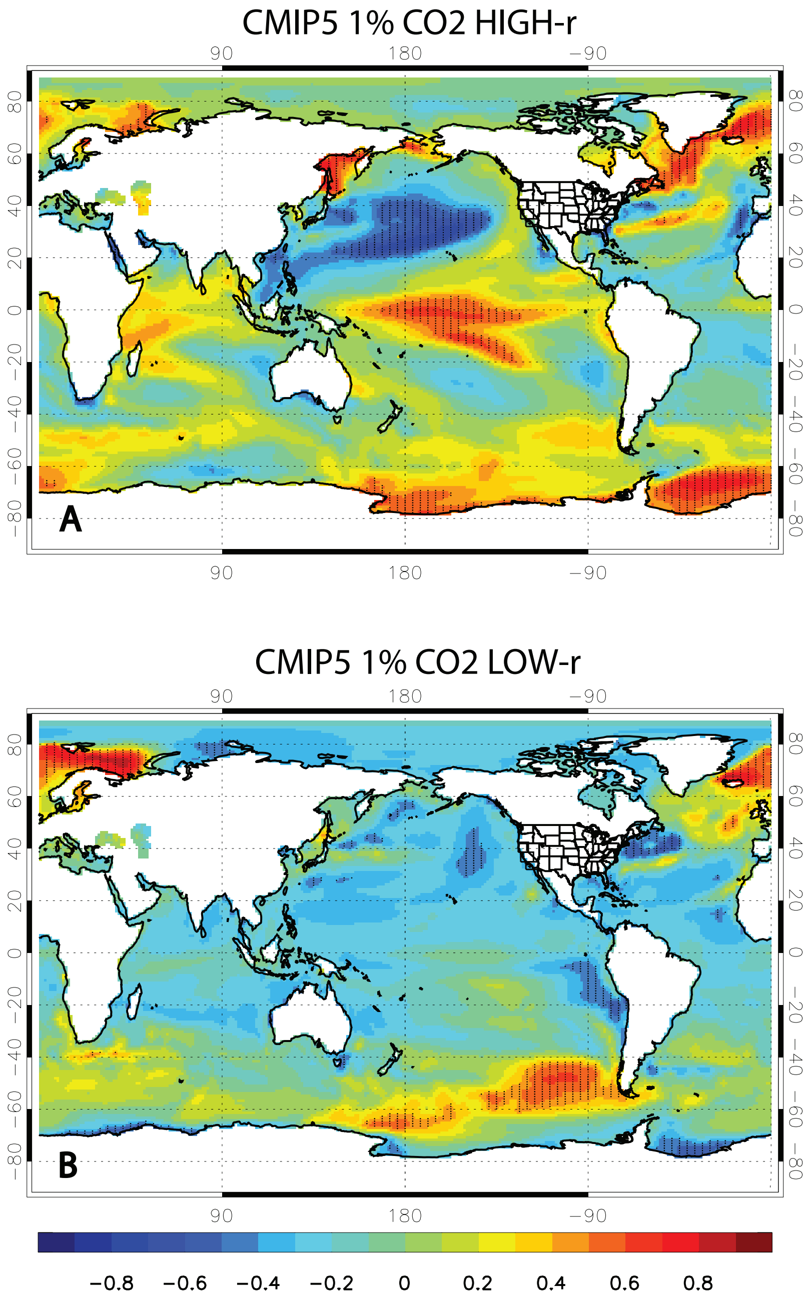

Supplementary Figure 6 **Coupled Model Intercomparison Project version 5 1% CO_2 December-January-February California precipitation versus sea surface temperature trend correlation map.** Trend correlation between CA precipitation and SSTs across individual realizations for (A) CMIP5 HIGH−r and (B) CMIP5 LOW−r using 1% CO_2 experiments. Symbols represent correlation significance at the 90% (diamond), 95% (X) or 99% (+) confidence level. Similar to CMIP5 RCP8.5 HIGH−r, 1% HIGH−r shows that larger CA precipitation trends are correlated with larger warming in the tropical Pacific. This further supports the importance of tropical Pacific SST warming to future increases in CA precipitation.

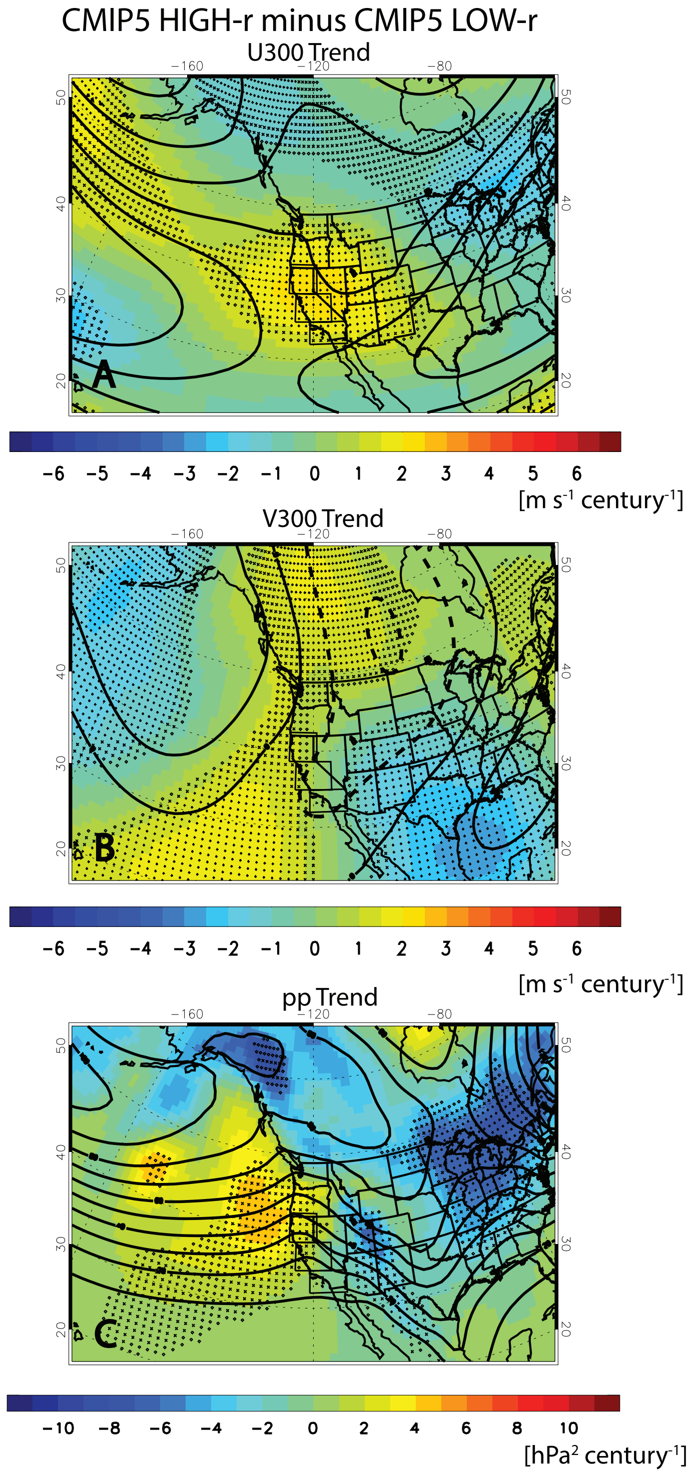

Supplementary Figure 7 **Coupled Model Intercomparison Project version 5 2006-2100 December-January-February extratropical dynamical changes.** (A) U300; (B) V300; and (C) *pp* trends for CMIP5 HIGH−r models minus CMIP5 LOW−r models. CMIP5 HIGH−r models yield a detrended DJF Niño 3.4 SST versus CA precipitation correlation of at least 0.30; CMIP5 LOW−r models yield a corresponding correlation less than 0.20. Trend units for winds are m s^{-1} $century^{-1}$; trend units for *pp* are hPa^2 $century^{-1}$. Symbols represent trend significance at the 90% (diamond), 95% (X) or 99% (+) confidence level, accounting for autocorrelation.

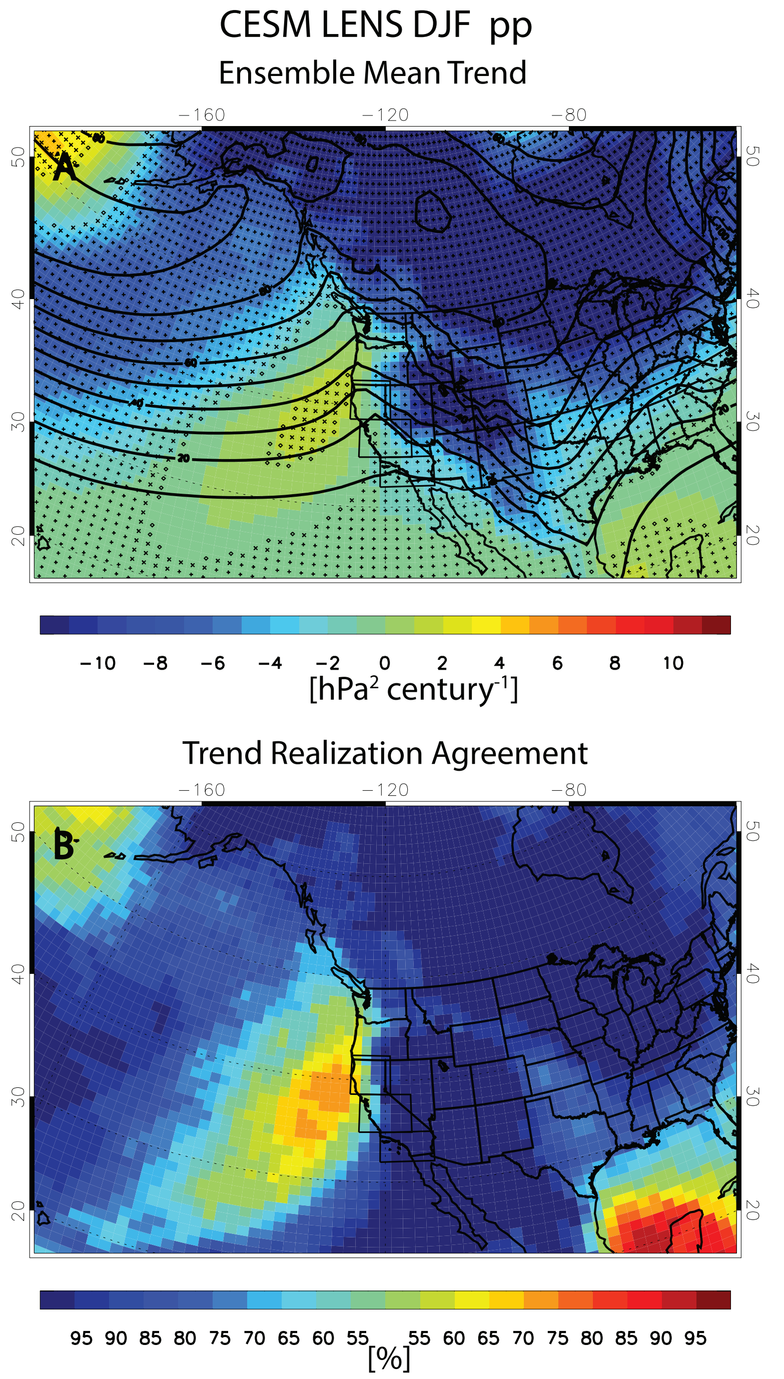

Supplementary Figure 8 **Community Earth System Model Large Ensemble 2006-2100 December-January-February storm track activity change.** A. Ensemble mean storm track activity (*pp*) trend [hPa^2 $century^{-1}$] and B. *pp* trend realization agreement. Warm (cold) colors in B. show the percent of realizations that yield an increase (decrease) in *pp*. Symbols in A. represent trend significance at the 90% (diamond), 95% (X) or 99% (+) confidence level, accounting for autocorrelation. Contour interval of climatological *pp* is 10 hPa^2.

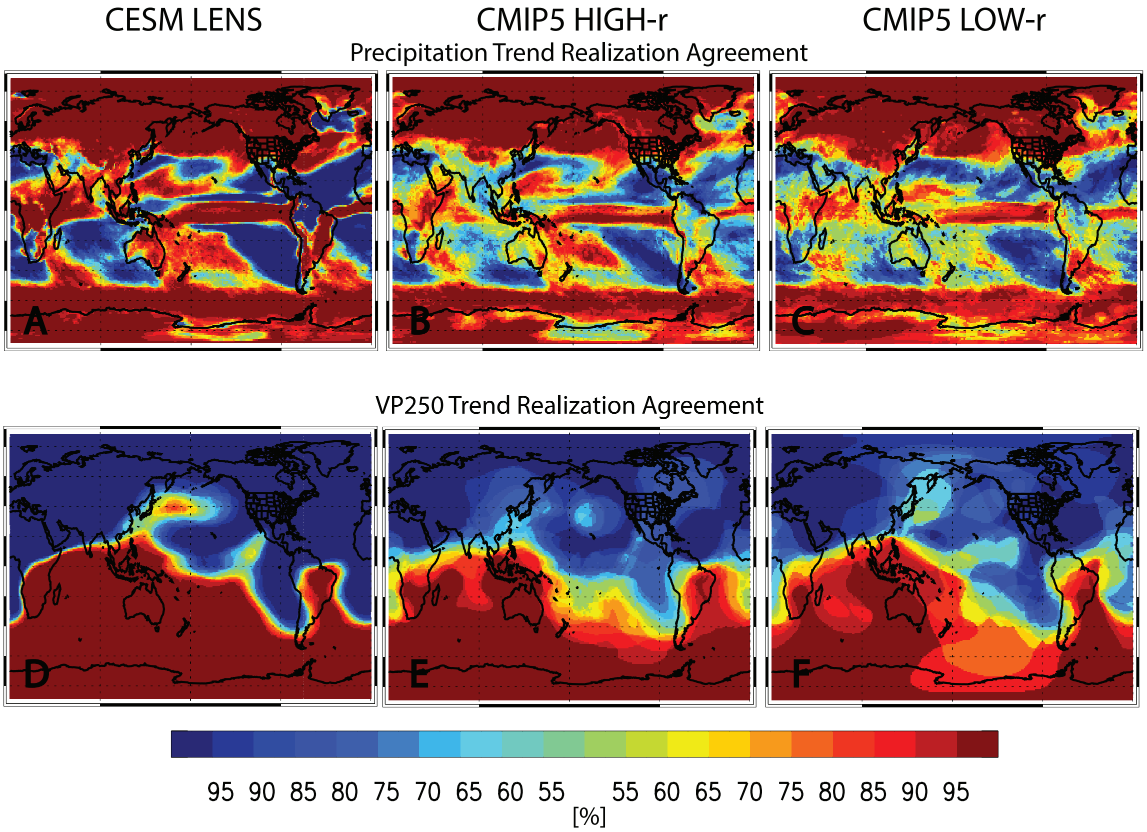

Supplementary Figure 9 **2006-2100 December-January-February trend realization agreement.** Trend realization agreement [%] for (A-C) precipitation; and (D-F) velocity potential at 250 hPa (VP250) for (left panels) CESM LENS, (center panels) CMIP5 HIGH−r and (right panels) CMIP5 LOW−r. Warm (cold) colors show the percent of realizations that yield an increase (decrease) in each quantity.

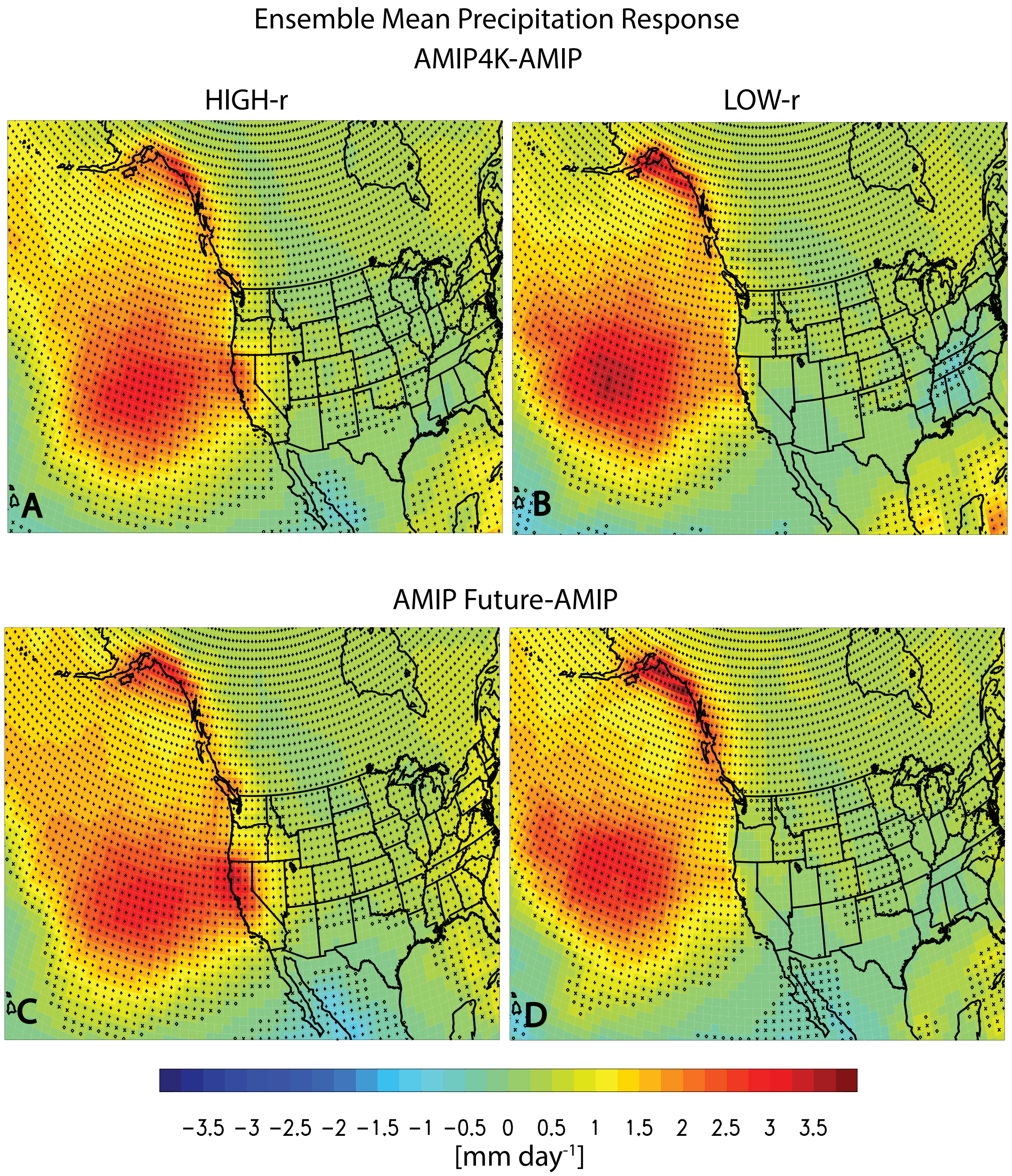

Supplementary Figure 10 **California precipitation response in atmosphere-only uniform and patterned sea surface temperature warming simulations.** Ensemble mean (top panels) uniform SST warming (AMIP4K−AMIP) and (bottom panels) patterned SST warming (AMIP Future−AMIP) precipitation response [mm day^{-1}] stratified by (left panels) HIGH−r and (right panels) LOW−r models. Symbols represent significance based on a t-test for the difference of means at the 90% (diamond), 95% (X) or 99% (+) confidence level.

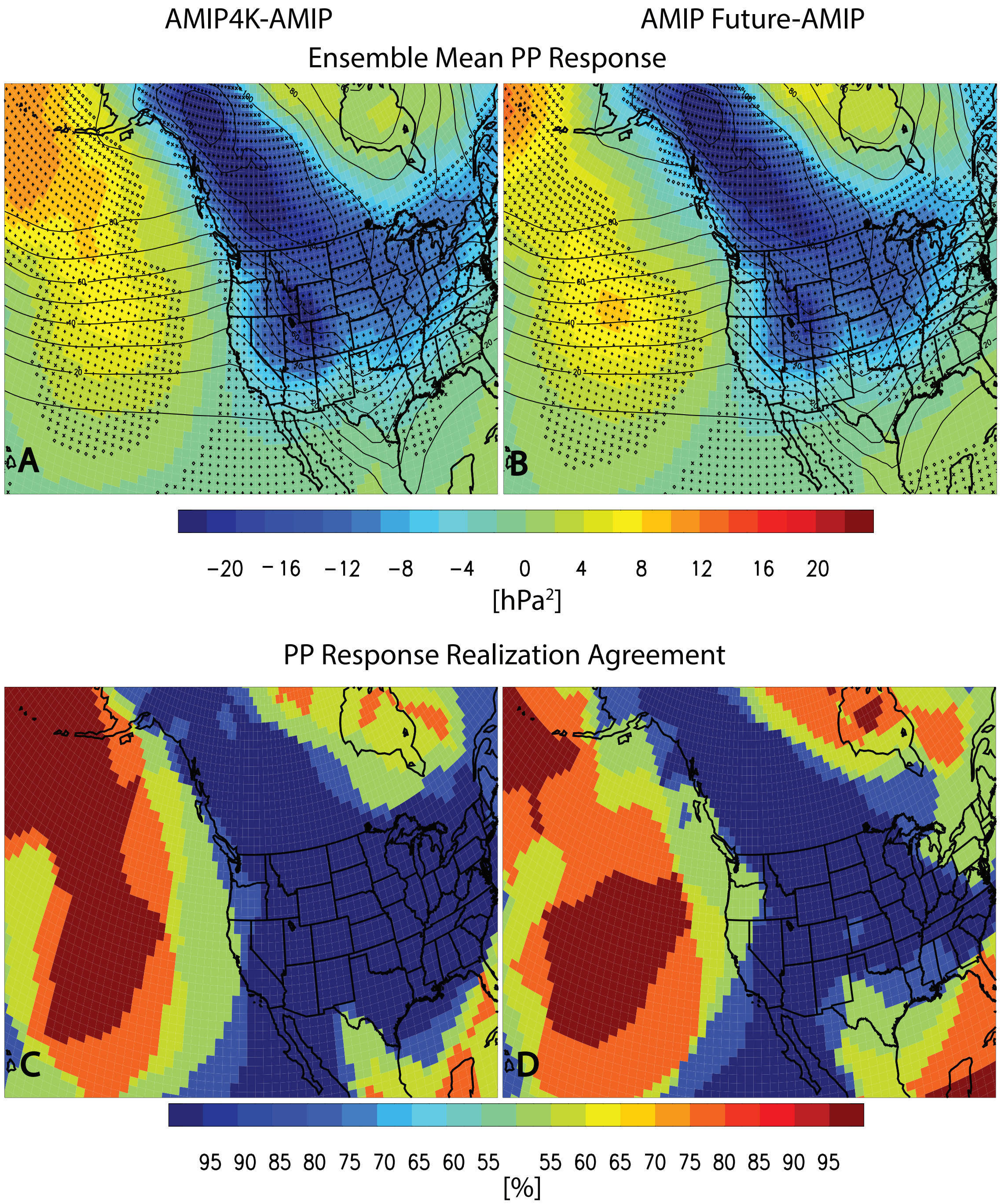

Supplementary Figure 11 **East Pacific storm track activity response in HIGH−r atmosphere-only uniform and patterned sea surface temperature warming simulations.** (A, B) Ensemble mean HIGH−r (left panels) uniform SST warming (AMIP4K−AMIP) and (right panels) patterned SST warming (AMIP Future−AMIP) *pp* response [hPa^2]. (C, D) shows the *pp* response realization agreement [%]. Symbols in (A, B) represent significance based on a *t*-test for the difference of means at the 90% (diamond), 95% (X) or 99% (+) confidence level.

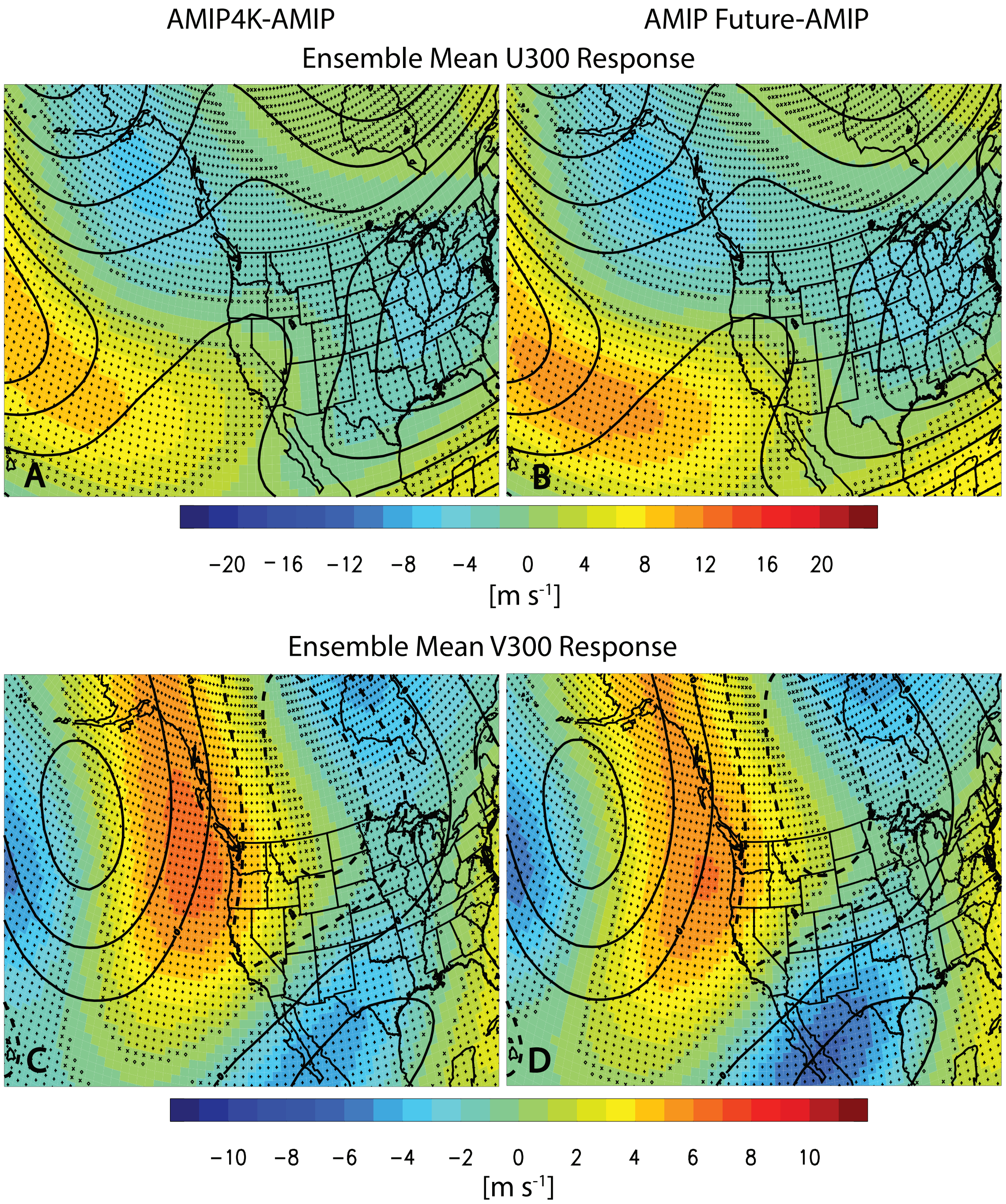

Supplementary Figure 12 **East Pacific 300 hPa zonal and meridional wind response in HIGH−r atmosphere-only uniform and patterned sea surface temperature warming simulations.** Ensemble mean HIGH−r (left panels) uniform SST warming (AMIP4K−AMIP) and (right panels) patterned SST warming (AMIP Future−AMIP) for 300 hPa (A, B) zonal winds (U300) and (C, D) meridional winds (V300). Symbols represent significance based on a *t*-test for the difference of means at the 90% (diamond), 95% (X) or 99% (+) confidence level. Climatological values are also included as thin black contour lines.

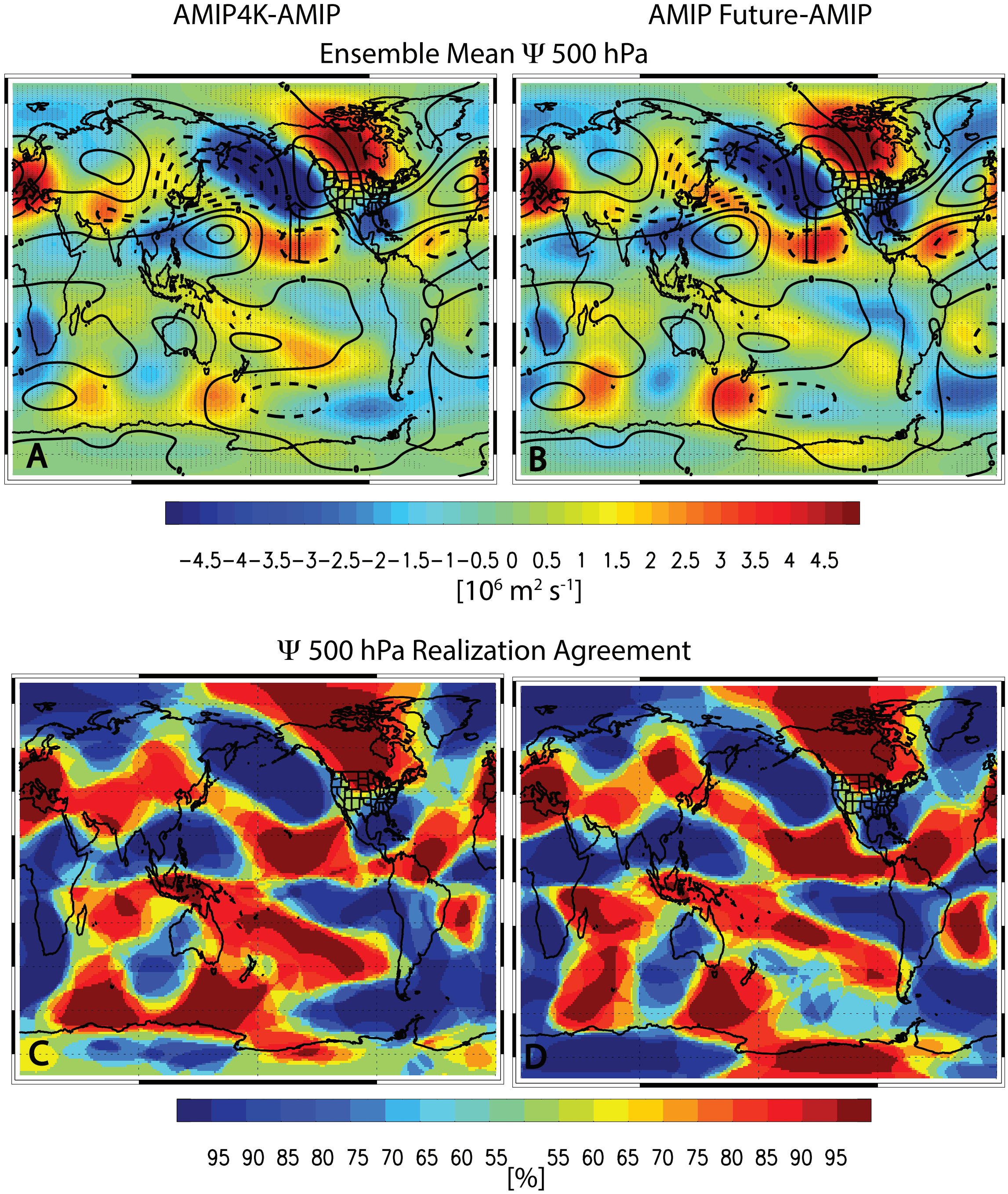

Supplementary Figure 13 **500 hPa stationary eddy stream function response in HIGH−r atmosphere-only uniform and patterned sea surface temperature warming simulations.** (A, B) Ensemble mean HIGH−r (left panels) uniform SST warming (AMIP4K−AMIP) and (right panels) patterned SST warming (AMIP Future−AMIP) 500 hPa stationary eddy stream function (Ψ500) response [10^6 m^2 s^{-1}]. (C, D) shows the Ψ500 response realization agreement [%]. Symbols in (A, B) represent significance based on a *t*-test for the difference of means at the 90% (diamond), 95% (X) or 99% (+) confidence level. Climatological values are also included as thin black contour lines. Black arrows sketch the direction in which the Rossby wave propagates in the Northern Hemisphere.

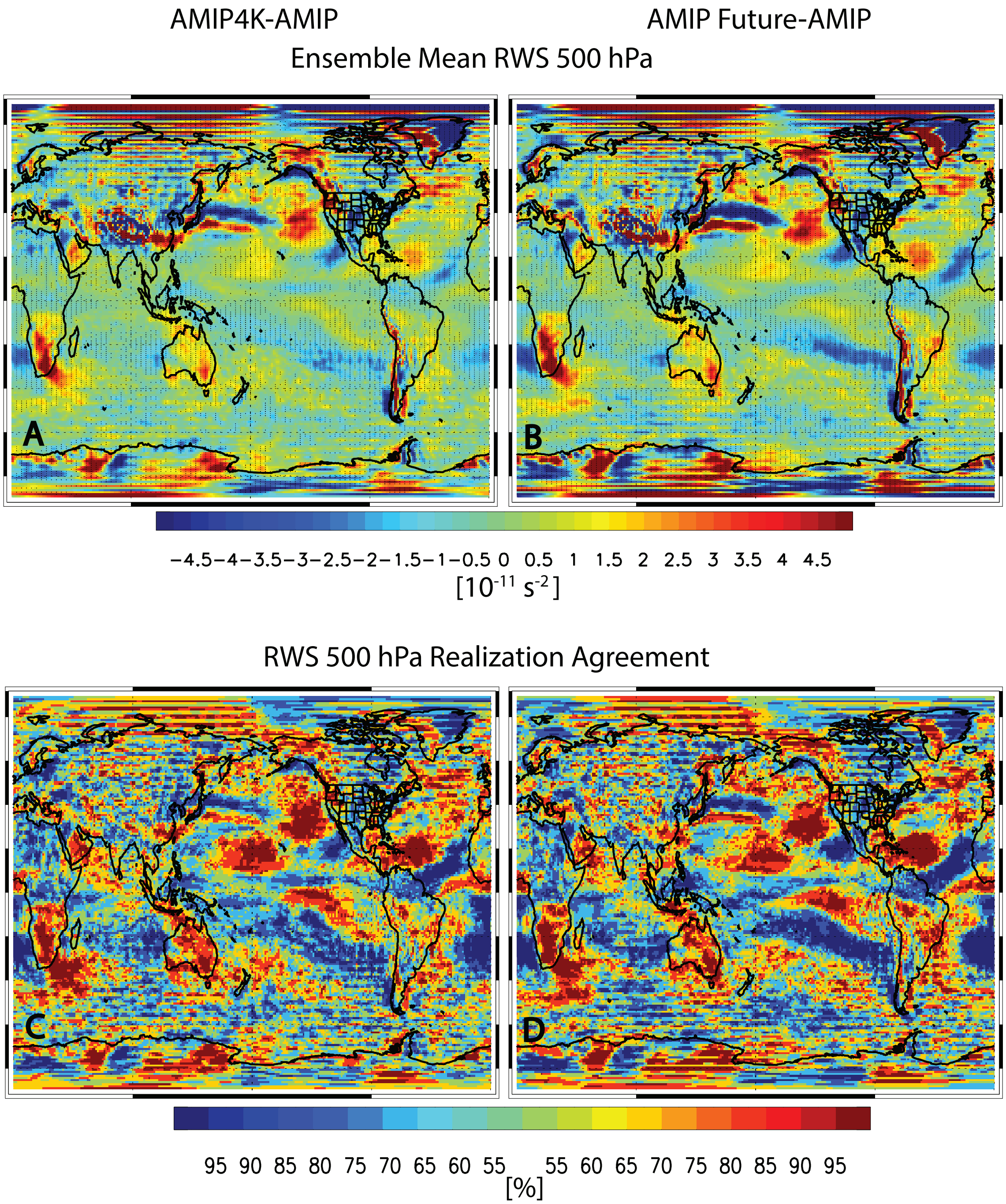

Supplementary Figure 14 **500 hPa Rossby wave source response in HIGH−r atmosphere only uniform and patterned sea surface temperature warming simulations.** (A, B) Ensemble mean HIGH−r (left panels) uniform SST warming (AMIP4K−AMIP) and (right panels) patterned SST warming (AMIP Future−AMIP) 500 hPa Rossby wave source (RWS 500) response [10^{-11} s^{-2}]. (C, D) shows the RWS 500 response realization agreement [%]. Symbols in (A, B) represent significance based on a t-test for the difference of means at the 90% (diamond), 95% (X) or 99% (+) confidence level.

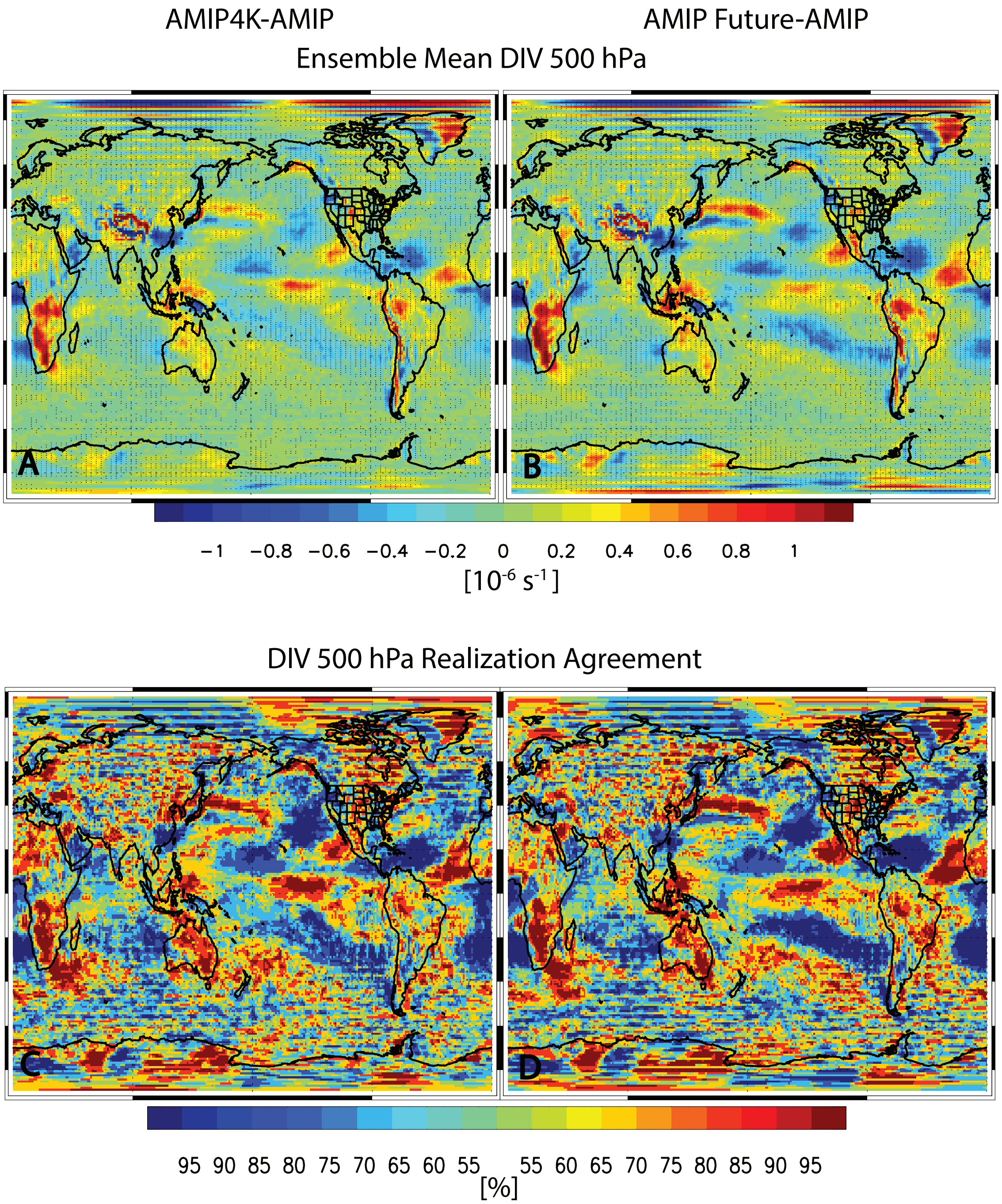

Supplementary Figure 15 **500 hPa divergence response in HIGH−r atmosphere-only uniform and patterned sea surface temperature warming simulations.** (A, B) Ensemble mean HIGH−r (left panels) uniform SST warming (AMIP4K−AMIP) and (right panels) patterned SST warming (AMIP Future−AMIP) 500 hPa divergence (DIV 500) response [10^{-6} s^{-1}]. (C, D) shows the DIV 500 response realization agreement [%]. Symbols in (A, B) represent significance based on a *t*-test for the difference of means at the 90% (diamond), 95% (X) or 99% (+) confidence level.

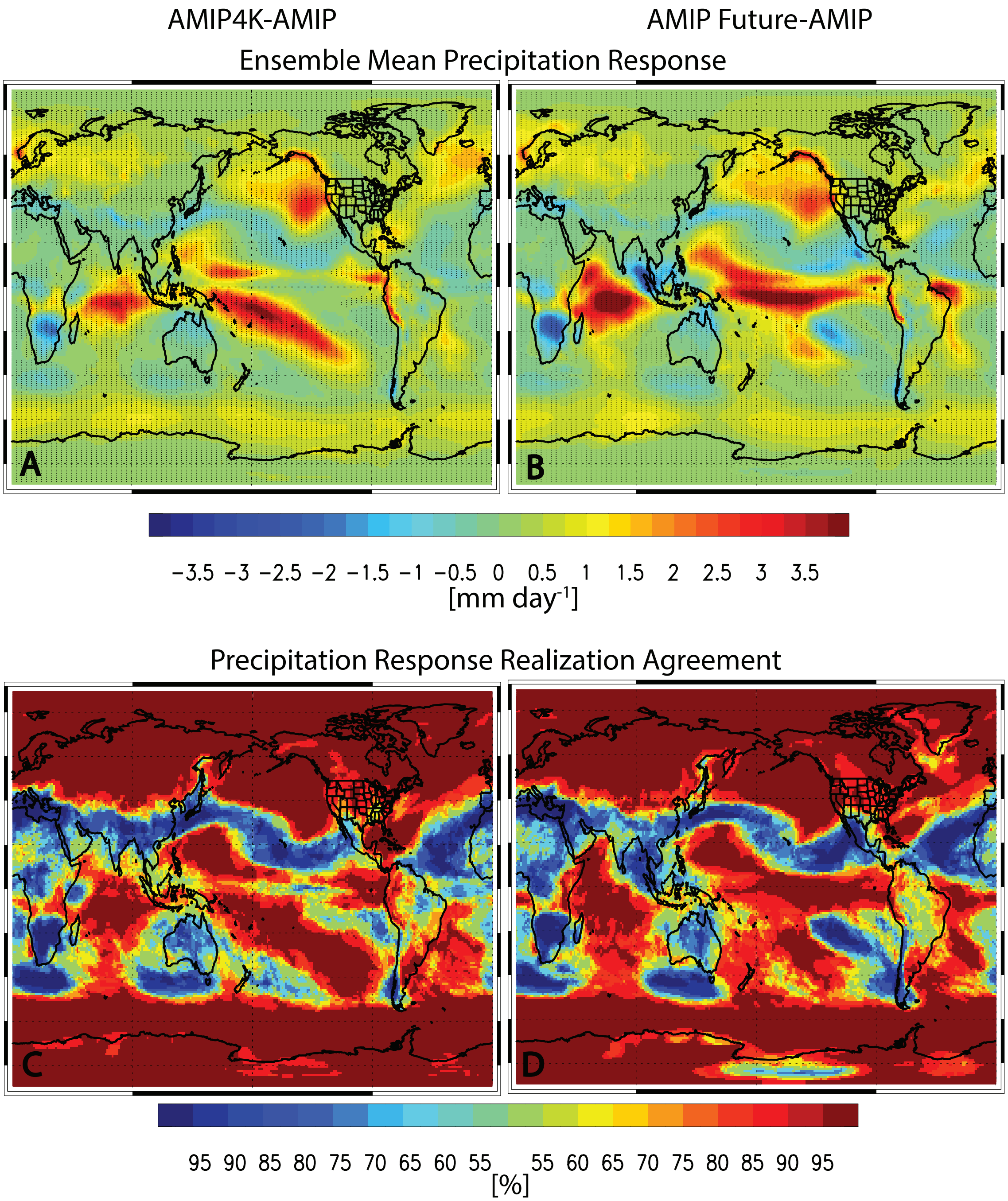

Supplementary Figure 16 **Precipitation response in atmosphere-only uniform and patterned sea surface temperature warming simulations.** (A, B) Ensemble mean HIGH−r (left panels) uniform SST warming (AMIP4K−AMIP) and (right panels) patterned SST warming (AMIP Future−AMIP) precipitation response [mm day^{-1}]. (C, D) shows the precipitation response realization agreement [%]. Symbols in (A, B) represent significance based on a t-test for the difference of means at the 90% (diamond), 95% (X) or 99% (+) confidence level.

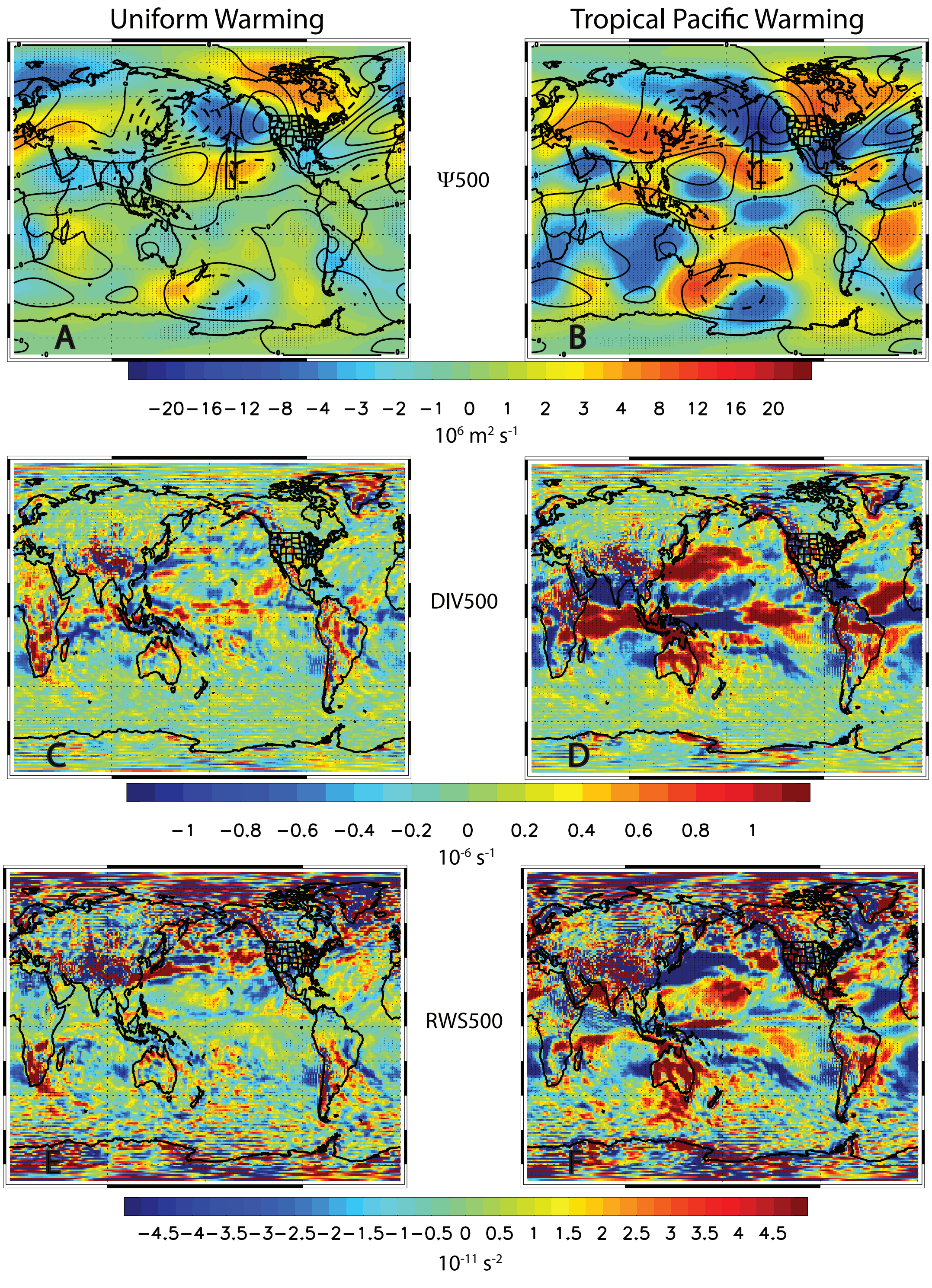

Supplementary Figure 17 **Community Atmosphere Model version 5 December-January-February uniform and tropical Pacific sea surface temperature warming tropical responses.** Uniform (left panels) and tropical Pacific (right panels) SST warming responses for (A, B) Ψ500 [10^6 m^2 s^{-1}]; (C, D) DIV500 [10^{-6} s^{-1}] and (E, F) RWS500 [10^{-11} s^{-2}]. Symbols represent significance based on a *t*-test for the difference of means at the 90% (diamond), 95% (X) or 99% (+) confidence level. Climatological values, and black arrows designating the direction in which the Rossby wave propagates in the Northern Hemisphere, are also included in (A-B).

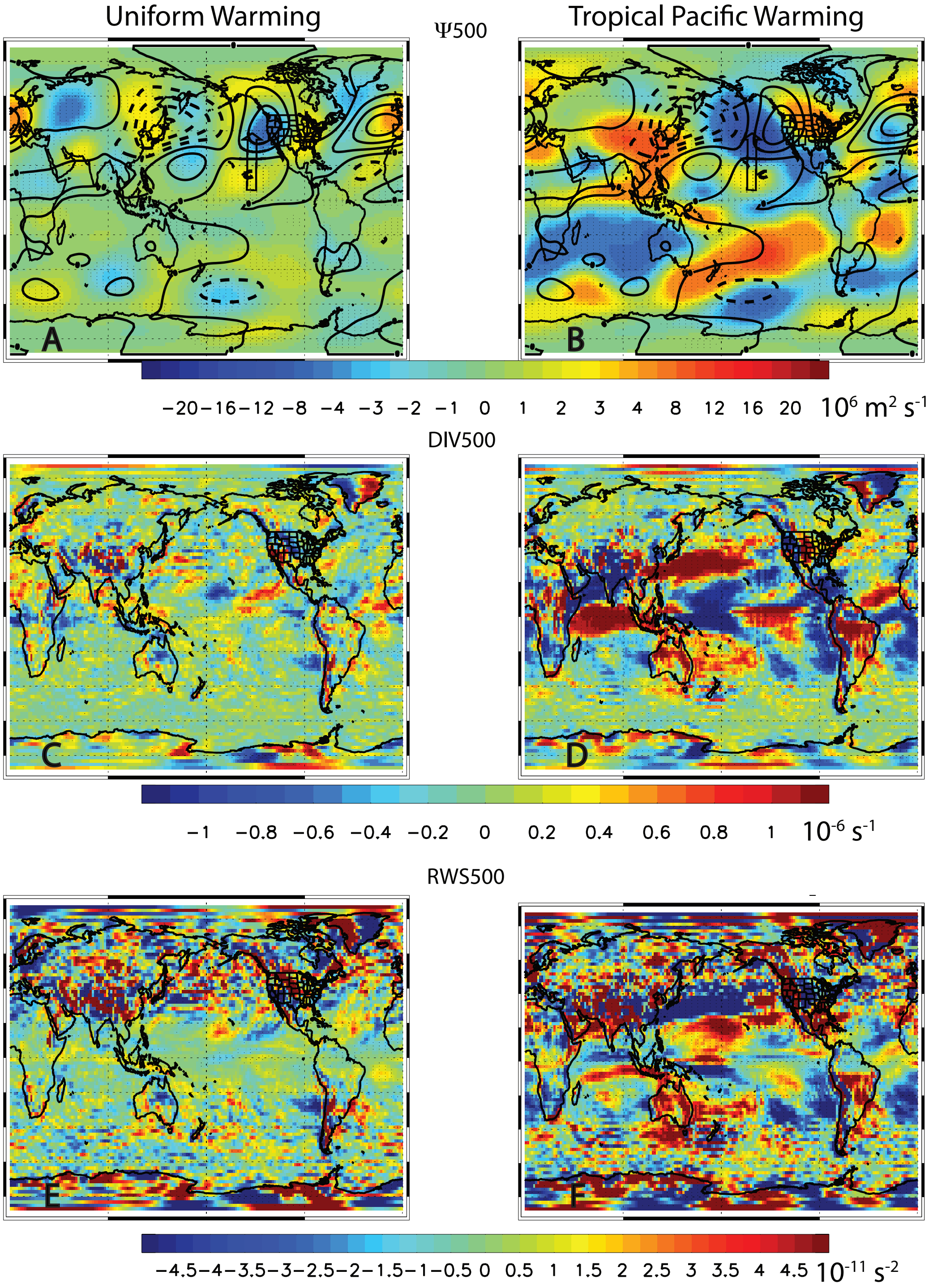

Supplementary Figure 18 **Geophysical Fluid Dynamics Laboratory Atmosphere Model version 3 December-January-February uniform and tropical Pacific sea surface temperature warming tropical responses.** Uniform (left panels) and tropical Pacific (right panels) SST warming responses for (A, B) Ψ500 [10^6 m^2 s^{-1}]; (C, D) DIV500 [10^{-6} s^{-1}] and (E, F) RWS500 [10^{-11} s^{-2}]. Symbols represent significance based on a *t*-test for the difference of means at the 90% (diamond), 95% (X) or 99% (+) confidence level. Climatological values, and black arrows designating the direction in which the Rossby wave propagates in the Northern Hemisphere, are also included in (A-B).

Supplementary Reference

Langenbrunner, B., and J. D. Neelin, 2013: Analyzing ENSO teleconnections in CMIP models as a measure of model fidelity in simulating precipitation. *J. Climate*, **26**, 4431– 4446, doi: 10.1175/JCLI–D–12–00 542.1.
